# Dentifrices for preventing erosive tooth wear: An in vitro comparative study

**DOI:** 10.1111/eos.70076

**Published:** 2026-02-24

**Authors:** Juliellen Luiz da Cunha, Anderson Gomes Forte, Elizabeth Barreto Galvão de Souza, Marcel Alves Avelino de Paiva, Débora Soares Bacelar, Fábio Correia Sampaio, Andressa Feitosa Bezerra de Oliveira

**Affiliations:** ^1^ Graduate Program in Dentistry Federal University of Paraíba (UFPB) João Pessoa Brazil; ^2^ Graduate Program in Dental Materials University of Campinas (UNICAMP) Piracicaba Brazil; ^3^ School of Dentistry Institutional Scientific Initiation Scholarship Program Federal University of Paraíba (UFPB) João Pessoa Brazil; ^4^ Department of Clinical and Social Dentistry Federal University of Paraíba (UFPB) João Pessoa Brazil; ^5^ Department of Morphology Federal University of Paraíba (UFPB) João Pessoa Brazil

**Keywords:** bioactive silica, fluoride dentifrices, stannous fluoride, tooth enamel

## Abstract

Erosive tooth wear is a progressive condition that compromises enamel structure and requires preventive strategies. This in vitro study aimed to compare the protective effects of fluoride‐based dentifrices containing various bioactive agents on sound enamel subjected to simulated erosive–abrasive challenges. Sixty bovine enamel blocks were randomly assigned to five dentifrices containing: bioactive silica + 1100 ppm F^−^ from sodium fluoride (BAS/NaF), 0.454% stannous fluoride (SnF), sodium‐calcium phosphosilicate + 1426 ppm F^−^ from sodium fluoride (SCP/NaF), 1100 ppm F^−^ from sodium fluoride (NaF), and a fluoride‐free (FF) control. Specimens underwent 7 days of pH cycling, including three daily acid challenges and 2‐min immersion in dentifrice slurries including 15 s brushing. Measurements included surface microhardness, surface roughness, fluorescence loss, and surface loss. Between‐group differences were assessed using one‐way anova with Tukey post hoc tests. The BAS/NaF and SnF dentifrices outperformed the other formulations, maintaining higher surface hardness and lower surface loss and fluorescence loss (*p* < 0.05). The NaF and the SCP/NaF dentifrices showed intermediate results. The fluoride‐free dentifrice exhibited the highest mineral loss. Dentifrices combining fluoride with bioactive ingredients enhanced enamel resistance to erosive–abrasive challenges, with bioactive silica and stannous fluoride showing the strongest effects.

## INTRODUCTION

Erosive tooth wear has become an increasingly relevant condition in modern dental practice, largely due to lifestyle factors such as frequent consumption of acidic foods and beverages, longer life expectancy, and extended tooth retention [1, 2, 3]. Recent epidemiological studies have reported rising prevalence rates of erosive tooth wear across all age groups, including adolescents, with early‐stage lesions detected as young as 13 years of age [4, 5, 6] or even younger. Erosive tooth wear is characterized by the irreversible loss of dental hard tissues caused by nonbacterial acids, resulting in progressive enamel demineralization and, in advanced stages, dentin exposure [1, 3, 7, 8]. This progression negatively impacts both functional and aesthetic aspects of teeth [8, 9]. The silent and often asymptomatic nature of erosive tooth wear hinders early diagnosis and leads to inadequate treatment [3, 10]. Given that natural protective mechanisms are limited under constant acid exposure, effective preventive strategies are essential [3, 6, 10, 11].

During the early stages of erosive tooth wear, acid exposure leads to partial hydroxyapatite dissolution, reducing enamel microhardness and increasing surface roughness, a process known as “superficial softening” [6, 10, 11, 12]. This softened layer of enamel becomes more vulnerable to brushing, accelerating enamel loss [7, 11]. In this context, toothpastes formulated to limit demineralization, enhance enamel resistance to both acids and combined erosion‐abrasion, and reduce hydrogen ion diffusion have shown promising results [13, 14, 15, 16].

Several formulations have been developed based on different mechanisms of action [10, 13, 14, 17, 18, 19, 20]. Advances in biomaterials engineering have enabled the creation of bioactive agents that not only protect enamel surfaces but also release remineralizing ions in a controlled manner [12, 13, 16, 17, 19, 21]. Toothpastes containing sodium fluoride (NaF) promote the deposition of CaF_2_‐like layers on enamel, acting as physical barriers to enhance remineralization in case of caries and to decrease demineralization in case of erosion [17]. Previous studies have shown that fluoride alone provided limited protection under repeated erosive–abrasive cycling [13, 14]. In contrast, stannous in combination with fluoride(SnF_2_)–based toothpastes have demonstrated superior performance by forming a protective layer of various compounds including stannous oxides, stannous phosphates and metal fluorides, which are more resistant to acid and abrasion [13, 18, 22, 23, 24]. Moreover, bioactive ingredients such as modified bioactive silica promote the formation of a biomimetic calcium phosphate silicate layer, which enhances enamel resistance to erosion [15, 16, 17]. Similarly, sodium–calcium phosphosilicate releases sodium, calcium, and phosphate ions in the presence of saliva, forming a hydroxyapatite‐like mineral layer that integrates with the enamel surface and enhances its resistance to acid attack [15, 16].

Given these advancements, this in vitro study aimed to compare the protective effects of fluoride‐based dentifrices containing bioactive agents on sound enamel subjected to simulated erosive–abrasive challenges. The study sought to determine the extent to which these formulations prevent mineral loss and preserve enamel surface integrity before the clinical manifestation of erosive tooth wear. The null hypotheses were: (1) that bioactive compounds combined with fluoride do not significantly affect enamel microhardness, surface roughness or fluorescence loss; and (2) that these compounds do not provide additional structural protection compared with a fluoride‐free control.

## MATERIAL AND METHODS

### Study design

In this in vitro study, bovine enamel blocks were randomly allocated to one of five interventions: four fluoride‐based dentifrices with or without bioactive ingredients and one fluoride‐free control. All specimens underwent 7 days of standardized erosion‐abrasion cycling. The predefined primary outcomes were surface microhardness change (ΔSH) and surface loss (SL), whereas fluorescence loss and surface roughness (Sa) were assessed as supportive parameters to characterize mineral and topographical alterations.

Surface microhardness was measured at baseline and after cycling, whereas surface roughness, fluorescence loss, and surface loss were assessed in the treated area and referenced to the adjacent sound area of each specimen.

### Sample selection

Sixty bovine enamel blocks were prepared from freshly extracted incisors obtained from a certified slaughterhouse as post‐mortem by‐products [25]. No animals were sacrificed for research, and use of animal‐derived material complied with institutional and international ethical standards. Teeth were stored in a 0.08% thymol‐buffered solution at room temperature for up to 30 days. Before use, each tooth was cleaned and examined under fivefold magnification using a stereomicroscope. Teeth with stains, cracks, fractures, or other enamel irregularities were excluded from the study.

Sample size was calculated based on Fernandes et al. [26] using the postcycling Vickers microhardness contrast between a bioactive‐silica + sodium fluoride dentifrice and a sodium‐calcium phosphosilicate (NovaMin) + sodium fluoride dentifrice (112.5 ± 27.3 vs. 81.8 ± 4.3 VHN). The between‐group mean difference was 30.7 VHN and the pooled within‐group SD 19.5 VHN, yielding Cohen's *d* = 1.57. Assuming *α* = 0.05 (two‐sided) and 80% power, the required sample size was 7 specimens per group; to allow for potential losses and ensure adequate power, we adopted *n* = 12 per group.

### Enamel block preparation

Sixty sound enamel blocks (4 × 4 × 2 mm) were prepared from the flattest region of the buccal surface of bovine incisor crowns and randomly assigned to five experimental groups (*n* = 12), according to the toothpaste tested. The teeth were obtained from postmortem by‐products of animals raised for commercial slaughter, in accordance with Brazilian legislation regulating animal experimentation (Law No. 11,794/2008, October 8, 2008). As animal experimentation is defined as procedures conducted on live animals, the use of animal‐derived tissues obtained postmortem does not require prior approval from an Ethics Committee on the Use of Animals. A precision cutting machine (Labcut 1010, Extec Corp.) with a double‐sided diamond disc was used, under constant water irrigation (approximately 200 mL/min), to cut the teeth into blocks. The blocks were embedded in acrylic resin and flattened using a metallographic polisher (PSK 2 V, Skilltec) with silicon carbide papers of increasing grit. Final polishing was performed with a 1 µm diamond suspension.

Baseline surface microhardness (SH_0_) was measured using a Vickers microhardness tester (HMV‐G21, Shimadzu). Three indentations were made at the center of each specimen, spaced 100 µm apart, with a 50 g load for 10 s. Only specimens with SH_0_ values within ±10% of the group mean (380 ± 10 VHN) were included. After SH_0_, the specimen edges were coated with two layers of acid‐resistant nail varnish (Risqué, Niasi) to create a protected reference area, leaving the central region exposed for experimental procedures.

### Toothpaste selection and preparation of slurries

Five different toothpaste formulations were tested, as described in Table 1. The selection aimed to represent distinct mechanisms of action and fluoride systems commonly found in consumer products. The following categories were included: (1) a bioactive silica + sodium fluoride formulation (REFIX Technology) designed to promote surface repair and mineral deposition; (2) a sodium–calcium phosphosilicate + sodium fluoride dentifrice (NovaMin) providing ionic release of calcium and phosphate for remineralization; (3) a stabilized stannous fluoride (SnF_2_) dentifrice with proven antierosive efficacy; (4) a conventional sodium fluoride (NaF) dentifrice serving as a fluoride‐only reference; and (5) a fluoride‐free formulation used as a negative control.

**TABLE 1:** Composition, main active ingredients, and manufacturers of the dentifrices tested.

Dentifrice technology	Technology	Ingredients	Fluoride concentration (ppm F^−^)	Manufacturer
Bioactive silica + sodium fluoride (BAS/NaF)	Acidified Bioactive Complex of Phosphate and Silica (REFIX Technology)	Sodium fluoride (NaF), acidified bioactive phosphate–silica complex, glycerin, hydrated silica, sorbitol, sodium lauryl sulfate, PEG‐12, cellulose gum, phosphoric acid, tetrasodium pyrophosphate, xylitol, cetylpyridinium chloride, sodium benzoate, sucralose, flavor, CI 16,255, limonene.	1.100	Rabbit Corporation
Sodium–calcium phosphosilicate + sodium fluoride (SCP/NaF)	NovaMin bioactive glass	Sodium Fluoride, Potassium Nitrate, Aqua, Sorbitol, Hydrated Silica, Glycerin, PEG, Cocamidopropyl Betaine, Aroma, Xanthan Gum, Sodium Saccharin, Titanium Dioxide (CI 77,891), Sodium Hydroxide, Limonene, Anisyl Alcohol.	1.426	Haleon plc
Stannous fluoride (SnF)	Stabilized SnF_2_ formulation	Stannous fluoride (SnF_2_), glycerin, hydrated silica, sodium hexametaphosphate, propylene glycol, PEG‐6, zinc lactate, sodium gluconate, sodium lauryl sulfate, flavor, trisodium phosphate, PVP, xanthan gum, carrageenan, CI 42,090.	1.100 (≈0.454% SnF_2_)	Procter & Gamble Company
Sodium fluoride (NaF)	Conventional NaF formulation	Sodium fluoride (NaF), sorbitol, water, glycerin, silica, sodium lauryl sulfate, sodium carboxymethylcellulose, flavor, xylitol, sodium saccharin, sodium benzoate.	1100	Rabbit Corporation
Fluoride‐free (FF)	Non‐fluoridated control formulation	Glycerin, sorbitol, water, silica, cellulose gum, flavor, xylitol, sodium saccharin, sodium benzoate. Detergent/surfactant: not disclosed.	_	Rabbit Corporation

*Note*: All formulations were commercially available toothpastes used as received. The fluoride concentrations are those declared by the manufacturers.

The study was designed to evaluate the potential synergistic effect of bioactive agents when combined with fluoride, rather than to isolate the action of the bioactive components. Therefore, only fluoride‐containing formulations and a fluoride‐free control were included, reflecting clinically relevant and commercially available toothpaste technologies. All NaF‐containing dentifrices had fluoride concentrations within the typical commercial range (≈1100–1450 ppm F^−^).

Each toothpaste was coded and transferred to an individual container by an independent researcher to ensure blinding. The dentifrices were identical in color, with all presenting a white appearance. The code was blinded to the examiner responsible for the experimental procedures and data analysis. Slurries were prepared daily by mixing each toothpaste with deionized water in a 1:3 ratio (w/w). The mixtures were homogenized under constant stirring for 4 min to ensure uniform consistency before each treatment.

### Cycling and erosive–abrasive treatments

**FIGURE 1:**
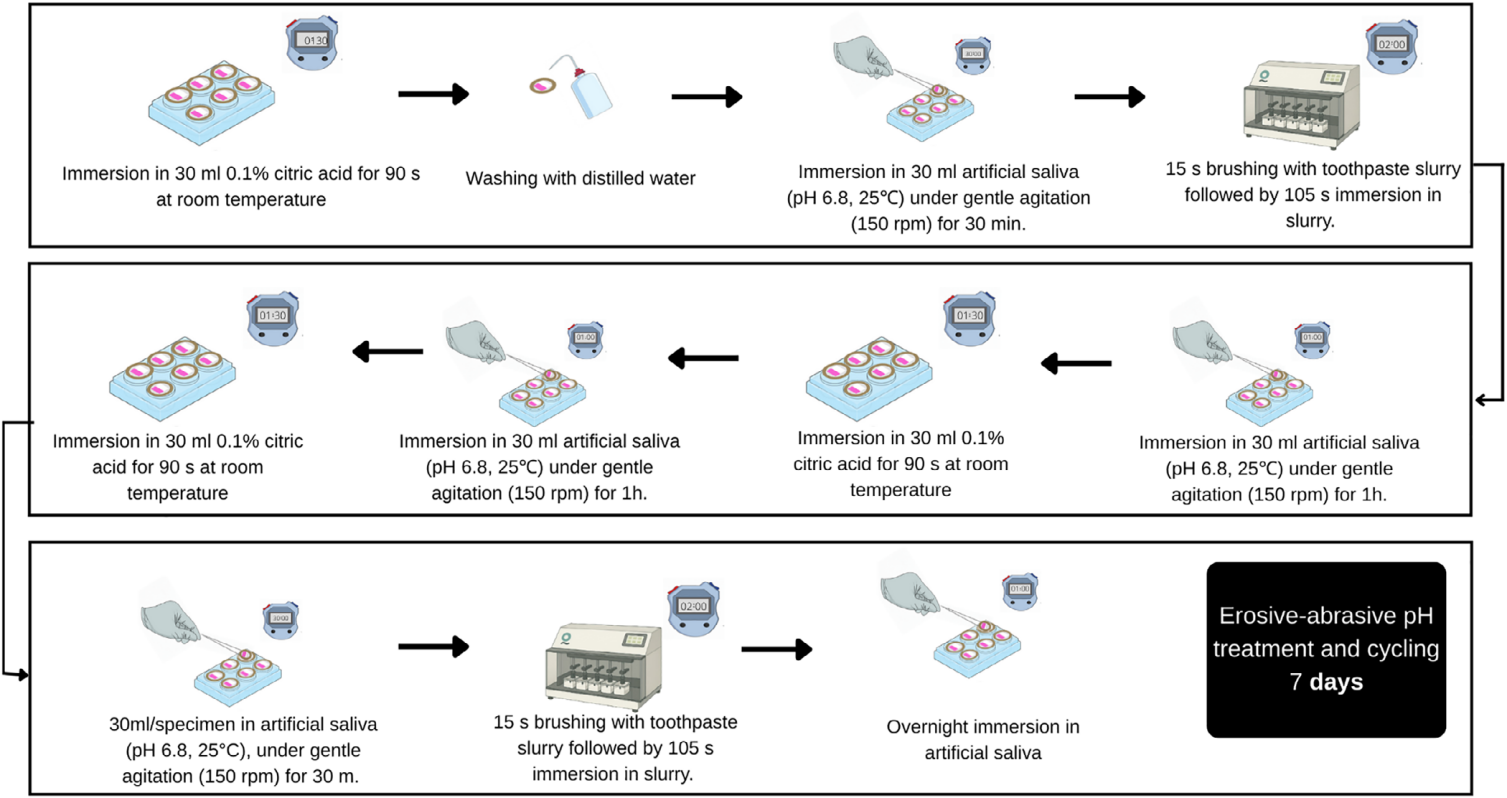
Schematic illustration of the 7‐day erosiveabrasive pH cycling protocol. Each daily cycle consisted of three erosive challenges (immersion in 0.1% citric acid, pH ≈ 2.5, for 90 s) alternating with immersion in artificial saliva (pH 6.8, 25°C) under gentle agitation (150 rpm). Brushing was performed three times per day for 15 s using dentifrice slurries (1:3 w/w), followed by 105 s additional immersion in slurry. Overnight immersion in artificial saliva simulated the resting phase. Total duration: 7 days.

The pH cycling protocol was adapted from Simões et al. [27] and Buzalaf et al. [12]. On Day 0, the specimens were stored overnight in artificial saliva. From Days 1 to 7, they underwent daily erosion‐abrasion cycles, as ilustrated in Figure 1.

The erosive challenge consisted of three daily immersions in 30 mL/sample of 0.1% citric acid solution (prepared in‐lab, pH 2.5) at room temperature (25°C) for 90 s without agitation. After each immersion, specimens were rinsed with deionized water for 5 s and stored in 30 mL/sample of artificial saliva (pH 6.8, 25°C) under gentle agitation (150 rpm) for 60 min. The artificial saliva contained 0.2 mM glucose, 9.9 mM NaCl, 1.5 mM CaCl_2_·2H_2_O, 3 mM NH_4_Cl, 17 mM KCl, 2 mM NaSCN, 2.4 mM K_2_HPO_4_, 3.3 mM urea, 2.4 mM NaH_2_PO_4_, and 11 µM ascorbic acid (prepared in‐lab, pH adjusted to 6.8).

After the first and final acid exposures, specimens were subjected to a subsequent 30‐min remineralization period and an abrasive challenge with standardized brushing. Each block was treated with 30 mL of toothpaste slurry (1:3 ratio, w/w) for 2 min: 15 s of brushing followed by 105 s immersion in slurry (no agitation). Brushing was performed using an automated device (MEV 3T‐8XY, Odeme) at 37°C. The brushing consisted of 11 cycles (15 s), with a vertical zigzag motion (25 mm amplitude in both directions) and an axial load of 150 g. A new soft‐bristled toothbrush was used for each specimen (Colgate Classic Clean Soft, Colgate‐Palmolive). After brushing, specimens were rinsed with deionized water and stored in artificial saliva until the next challenge.

During the overnight period, specimens remained immersed in artificial saliva. All solutions were freshly prepared; the 0.1% citric acid was renewed before each immersion, and the artificial saliva was replaced daily.

### Surface microhardness analysis

Surface microhardness was measured at baseline (SH_0_) and after the experimental cycling (SH_1_), as previously described. All measurements were performed by a calibrated examiner. The percentage of surface microhardness change (%SMHC = [(SH_0_ − SH_1_)/SH_0_] × 100) was also calculated to account for baseline variability and improve comparability among groups.

### Quantitative light‐induced fluorescence analysis

Quantitative light‐induced fluorescence (QLF) was used as a nondestructive and sensitive optical method to quantify mineral changes on enamel surfaces [28, 29]. QLF images were acquired with Qraycam Pro (Qraycam Pro, Inspektor Research Systems BV) and analyzed in QA2 software (version 1.38, Inspektor Research Systems BV). Before imaging, the nail varnish was carefully removed with cotton swabs moistened with diluted acetone, followed by rinsing with deionized water and air‐drying. A standardized setup was used to ensure reproducible image acquisition: the camera was fixed at an 8‐cm distance in a dark room, with exposure and contrast set to zero for all images. The region of interest (ROI) was delineated, and the fluorescence intensity within the exposed area was compared with that of adjacent sound enamel.

Mean fluorescence loss (given in %) was defined as the average percentage decrease in fluorescence within the ROI relative to adjacent sound enamel, reflecting the overall extent of mineral loss. Maximum fluorescence loss (given in %) represented the greatest local decrease in fluorescence. However, in erosive–abrasive models, maximum fluorescence loss should not be interpreted as a direct indicator of lesion depth, as fluorescence attenuation may also arise from surface roughness and light scattering rather than true volumetric loss [25, 26]. Accordingly, fluorescence loss was used as the primary fluorescence parameter for between‐group comparisons, whereas maximum fluorescence loss was retained only as an exploratory descriptor, consistent with current. Accordingly, QLF was applied in accordance with current recommendations for erosive tooth wear assessment, using fluorescence loss as a parameter with demonstrated accuracy and precision for enamel mineral evaluation [30, 31, 32].

### Surface profilometry analysis

Surface morphology was assessed using a noncontact 3D optical profilometer (Talysurf CCI MP, Taylor Hobson). The analysis was performed with a 20× magnification, a 0.86 × 0.86 mm^2^ field of view, and XY scanning mode at 1024 × 1024 px resolution. A low‐reflectance setting (level 4), 0.25 mm cut‐off, and Gaussian filter (ISO 16,610–61) were applied to the height data; the filtered data were used to calculate surface loss (SL, µm) and surface roughness (Sa, µm).

Surface roughness (Sa, µm) was defined as the arithmetical mean height of the measured area. Readings were obtained on the sound reference region (Sa_0_) and on the treated region (Sa_1_); the difference in roughness was calculated as ΔSa = Sa_1_ − Sa_0_ (negative values indicate surface smoothing).

Surface loss (µm) was defined as the vertical height difference between the varnish‐protected reference area and the exposed central region. Height profiles were recorded along three predetermined lines (25%, 50%, and 75% of the specimen length), and the mean of these readings was used to calculate surface loss.

Representative 3D images were generated to illustrate surface loss and roughness in each group.

### Data analysis

Data were analyzed using SPSS software (version 21.0). Shapiro–Wilk was used to assess the normality of the data, and Levene's test was used to check homogeneity of variances. Since both assumptions were met, no data transformation was required. For between‐group comparisons, one‐way anova with Tukey's post hoc for was used: surface microhardness (SH_0_; SH_1_, %SMHC), fluorescence‐loss readings (fluorescence loss in %; maximum fluorescence loss in %), surface roughness (Sa_0_; Sa_1_, µm), and surface loss (µm). Paired *t*‐tests were applied for intragroup (pre‐post) comparisons (SH_0_ vs. SH_1_; Sa_0_ vs. Sa_1_). Results are reported as point estimates (means/mean differences) with 95% confidence intervals; statistical significance was set at *α* = 0.05.

## RESULTS

Surface microhardness values are presented in Table 2. At baseline (SH_0_), no statistically significant differences were observed between groups, confirming proper sample standardization. After treatment (SH_1_), the fluoride‐free group exhibited a significantly greater hardness loss than all other groups (*p* < 0.05). The sodium fluoride (NaF) and sodium–calcium phosphosilicate + sodium fluoride (SCP/NaF) dentifrices did not differ from each other (95% CI: −10.2 to 6.1). The bioactive silica + sodium fluoride (BAS/NaF) and stannous fluoride (SnF) dentifrices presented the highest final microhardness values, with no significant difference between them (95% CI: −9.5 to 5.9) both outperformed NaF, SCP/NaF, and the fluoride‐free control (*p* < 0.05).

**TABLE 2:** Mean values (±SD) of surface microhardness (SH, Vickers hardness) at baseline (SH_0_) and after erosive–Abrasive cycling (SH_1_), and percentage of surface microhardness change (%SMHC) for each dentifrice group.

Dentifrices	SH_0_	SH_1_	%SMHC
Bioactive silica + sodium fluoride	387.9 ± 2.0^a,A^	288.5 ± 6.2^a,B^	26.8 ± 1.3^a^
Sodium–calcium phosphosilicate + sodium fluoride	385.6 ± 2.3^a,A^	227.1 ± 6.2^b,B^	41.0 ± 1.6^b^
Stannous fluoride	388.5 ± 2.7^a,A^	290.0 ± 12.3^a,B^	25.0 ± 3.2^a^
Sodium fluoride	389.5 ± 1.3^a,A^	221.5 ± 7.8^b,B^	43.1 ± 1.9^b^
Fluoride‐free	386.2 ± 2.6^a,A^	160.2 ± 4.5^c,B^	58.7 ± 1.0^c^

*Note*: Different lowercase letters indicate statistically significant between‐group differences within a column (one‐way anova with Tukey post hoc test, *α* = 0.05). Different uppercase letters indicate statistically significant within‐group differences between SH_0_ and SH_1_ (paired *t*‐test, *α* = 0.05).

The percentage of microhardness change (%SMHC) followed a similar pattern found for SH_1_. The BAS/NaF and SnF groups exhibited the lowest %SMHC values (95% CI: −5.9 to 9.5), without significant difference between them, and were significantly more effective than the other dentifrices. The fluoride‐free group had the highest %SMHC, differing significantly from all others (*p* < 0.05).

Table 3 presents the fluorescence data. The BAS/NaF and SnF groups showed the lowest fluorescence loss values, with no statistically significant difference between them (95% CI: −1.21 to 1.41), and were significantly lower than those of all other groups (*p* < 0.05). The FF dentifrice showed the highest fluorescence loss, differing significantly from all others (*p* < 0.05). A similar pattern was observed for maximum fluorescence loss. The BAS/NaF and SnF groups did not differ significantly from each other (95% CI: −2.1 to 1.9) and both showed lower values than the fluoride‐free control. No significant differences were detected between the NaF and SCP/NaF groups for either fluorescence loss (95% CI: −0.2 to 2.5) or maximum fluorescence loss (95% CI: −0.8 to 3.4).

**TABLE 3:** Fluorescence loss (ΔF, %) and maximum fluorescence loss (ΔF_max_, %) assessed by quantitative light‐induced fluorescence (QLF).

Dentifrices	ΔF	ΔF_max_
Bioactive silica + sodium fluoride	−6.33 ± 0.62^c^	−7.46 ± 0.95^c^
Sodium–calcium phosphosilicate + sodium fluoride	−9.41 ± 0.79^a^	−15.23 ± 1.46^a^
Stannous fluoride	−5.97 ± 0.70^c^	−7.25 ± 1.88^c^
Sodium fluoride	−10.53 ± 0.54^a^	−16.55 ± 1.19^a^
Fluoride‐free	−15.87 ± 1.71^b^	−21.54 ± 1.51^b^

*Note*: Values represent mean ± standard deviation (SD). Different lowercase letters indicate statistically significant between‐group differences within each column (one‐way anova with Tukey post hoc test, *α* = 0.05).

Surface roughness data (Table 4) showed no significant baseline differences (Sa_0_) among groups. After treatment (Sa_1_), BAS/NaF and SnF exhibited similar roughness values (95% CI: −0.2 to 0.2). NaF and fluoride‐free groups also showed no statistically significant differences (95% CI: −0.5 to 0.07). The SCP/NaF group presented significantly higher Sa_1_ values than all other formulations (*p* < 0.05).

**TABLE 4:** Mean (±SD) 3D profilometry outcomes: Surface roughness on sound (Sa_0_, µm) and treated (Sa_1_, µm) areas, difference in surface roughness (ΔSa, µm), and surface loss (SL, µm).

Dentifrices	Sa_0_	Sa_1_	ΔSa	SL
Bioactive silica + sodium fluoride	0.0461 ± 0.019^a,A^	0.2100 ± 0.048^a,B^	0.1638 ± 0.046^a^	2.88 ± 0.60^a^
Sodium–calcium phosphosilicate + sodium fluoride	0.0602 ± 0.020^a,A^	0.6525 ± 0.286^b,B^	1.032 ± 0.190^b^	5.11 ± 0.82^b^
Stannous fluoride	0.0591 ± 0.025^a,A^	0.2437 ± 0.038^a,B^	0.1603 ± 0.060^a^	2.81 ± 0.59^a^
Sodium fluoride	0.0454 ± 0.018^a,A^	0.994 ± 0.082^c,B^	0.9486 ± 0.068^a,b^	5.7 ± 0.80^b^
Fluoride‐free	0.0470 ± 0.013^a,A^	1.250 ± 0.135^c,B^	1.203 ± 0.124^b^	10.5 ± 1.36^c^

*Note*: Different lowercase letters indicate statistically significant between‐group differences within a column (one‐way anova with Tukey post hoc test, *α* = 0.05). Different uppercase letters indicate statistically significant within‐group pre–post differences (paired *t*‐test, *α* = 0.05).

Regarding ΔSa, BAS/NaF and SnF exhibited the lowest values and did not differ from each other (95% CI: −0.73 to 0.73). SCP/NaF and fluoride‐free dentifrice showed the highest ΔSa values, also without significant difference between them (95% CI: −0.69 to 1.03). The NaF dentifrice showed intermediate ΔSa values and did not differ significantly from any of the other groups (*p* > 0.05).

The results for enamel surface loss are shown in Table 4. The SnF dentifrice produced the lowest surface loss values, followed closely by BAS/NaF (95% CI: –0.98 to 1.14). Both were significantly more effective in minimizing surface loss than SCP/NaF, NaF, and fluoride‐free groups (*p* < 0.05). The NaF and SCP/NaF exhibited intermediate performance (95% CI: −1.8 to 0.6). The fluoride‐free group showed the greatest surface loss, differing from all other formulations (*p* < 0.05).

**FIGURE 2:**
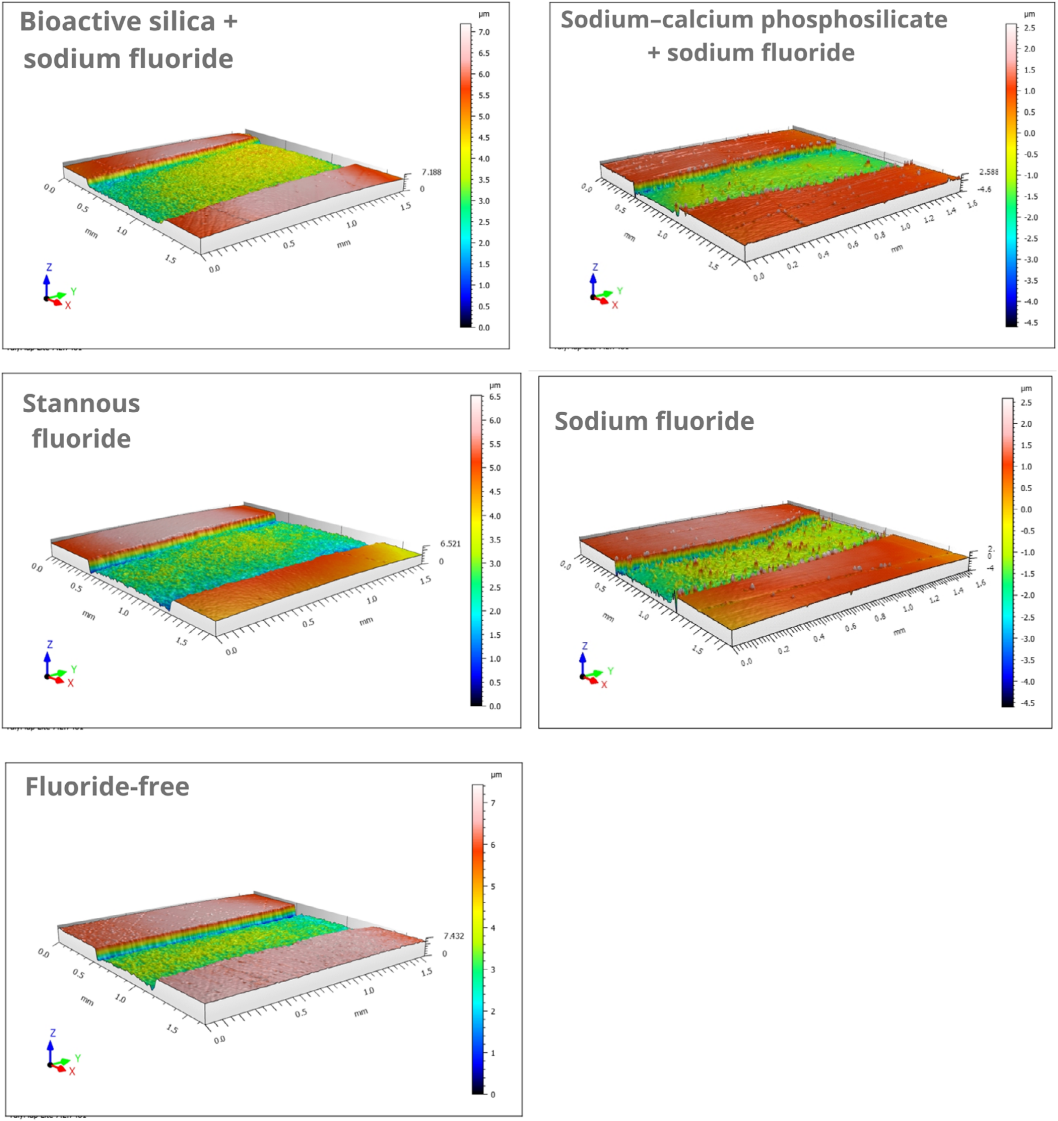
Optical profilometry images of enamel surfaces after 7 days of erosive–abrasive cycling. The color scale represents surface height (µm), where blue indicates depressions and red indicates elevated regions. All panels share identical scanning parameters (*z*‐range −2.5 to +2.5 µm), except the fluoride‐free control, which required an expanded *z*‐range (0–7.0 µm) due to higher surface loss.

Representative 3D profilometric reconstructions (Figure 2) corroborated these quantitative results. Specimens treated with SnF and BAS/NaF dentifrices exhibited well‐preserved surface contours with minimal depressions, consistent with their low surface loss values. SCP/NaF and NaF showed localized wear features corresponding to intermediate surface loss, whereas the fluoride‐free specimens displayed pronounced depressions and irregular topography, in line with the highest surface loss values.

## DISCUSSION

This in vitro study examined the protective effect of fluoride‐based toothpastes containing different bioactive agents on sound enamel subjected to simulated erosive–abrasive cycling. The null hypotheses were partially rejected, as both the presence and type of active ingredient significantly influenced enamel preservation. Dentifrices containing stannous fluoride and bioactive silica provided superior protection compared to the conventional sodium fluoride and the fluoride‐free dentifrices. Dentifrices containing stannous fluoride or bioactive silica provided superior protection, compared with conventional sodium fluoride and the fluoride‐free control [12, 33, 34, 35].

Bioactive agents have been shown to promote surface mineral recovery and stabilization of the partially demineralized enamel under both cariogenic and erosive conditions [6, 12, 33, 34]. Surface microhardness was selected as a primary outcome due to its sensitivity to early mineral loss [16, 29], although postchallenge values also reflect the removal of acid‐softened enamel during brushing [29]. As erosive–abrasive challenges may result in partial surface loss and affect the thickness of the remaining subsurface softened enamel layer, post‐treatment surface microhardness was interpreted as an indicator of the mechanical resistance of the residual enamel surface rather than as a measure of remineralization. To ensure a comprehensive interpretation, surface microhardness findings were evaluated together with surface loss, surface roughness (Sa), and fluorescence loss. Surface roughness provided insight into early morphological alterations [10, 36], while quantitative light‐induced fluorescence offered a nondestructive measure of mineral integrity [28]. In line with recent recommendations [28, 29, 36], fluorescence loss was used as the primary fluorescence metric, and maximum fluorescence loss was retained only as a secondary descriptive parameter. This multimodal approach strengthened the overall interpretation of enamel response to erosive–abrasive challenges.

The erosive–abrasive protocol incorporated sequential acidic challenges followed by a 30‐min immersion in artificial saliva prior to brushing. Although delaying brushing does not consistently reduce erosive tooth wear compared with immediate brushing [37], the interval standardized the design and allowed partial surface coverage by mineral precipitates. This approach has been adopted in previous in vitro studies to ensure controlled conditions and to provide a conservative and reproducible assessment of the protective effect of antierosive toothpastes following an acidic challenge [25, 38].

The superior performance of BAS/NaF and SnF suggests that combining fluoride with ion‐releasing or surface‐reactive agents enhances enamel resistance to both acid demineralization and abrasion [12, 15, 16, 22, 23, 24]. Such formulations may be especially beneficial for individuals with high exposure to acidic beverages or vigorous brushing habits.

The stabilized stannous fluoride dentifrice demonstrated strong protective effects, maintaining higher microhardness, reducing fluorescence loss, and minimizing surface loss. These benefits are associated with the formation of a tin‐rich surface layer that decreases enamel solubility and increases resistance to acid challenges and abrasion [18, 19, 34, 39, 40, 41]. In this in vitro model, protection is primarily attributed to direct stannous deposition on enamel, since pellicle‐mediated effects require salivary proteins absent from the artificial saliva [19, 39, 40, 41]. Under in situ conditions, however, Sn^2+^ ions may also bind to organic components of the acquired pellicle, further strengthening the protective barrier [22, 23, 24]. These mechanisms align with previous evidence showing enhanced enamel resistance with stabilized stannous‐containing dentifrices [18, 19, 39, 40, 41].

The bioactive silica + sodium fluoride dentifrice (BAS/NaF) also effectively reduced enamel loss. This formulation is based on an ion‐releasing bioactive silica associated with fluoride, which promotes the release of calcium and phosphate ions and the formation of a calcium–phosphate–silica‐rich surface layer on enamel, increasing its resistance to erosive and abrasive challenges [12, 15, 16, 20, 35, 42]. Similar ion‐mediated mechanisms have been shown to enhance surface microhardness and reduce enamel surface loss by forming a protective mineral layer, as demonstrated in recent in vitro studies [15, 16, 29, 43, 44]. These findings support the capacity of ion‐releasing bioactive systems to complement fluoride action under erosive–abrasive conditions [12, 15, 16].

The fluoride‐free dentifrice consistently presented the poorest performance across all evaluated parameters. The absence of active agents capable of promoting surface mineral deposition or forming protective surface layers resulted in substantially greater enamel loss and roughness compared with all other formulations [14, 16, 19, 20, 27]. This reinforces the central roles of fluoride and bioactive compounds in mitigating erosive–abrasive damage [12, 15, 19], particularly in patients at increased risk of erosion [16, 31].

Although fluoride is well established in caries prevention, its isolated action appears less effective under repeated erosive–abrasive cycling, a finding consistent with previous reports [14, 16, 20, 33]. Reduced efficacy is partly explained by limited fluoride retention under highly demineralizing conditions, which restricts the formation of calcium fluoride‐like reservoirs [11, 12, 13, 33, 45]. Consequently, conventional NaF formulations may offer only partial protection, underscoring the relevance of delivery systems or bioactive ingredients capable of enhancing fluoride retention and bioavailability [16, 20, 21, 35, 46].

The sodium‐calcium phosphosilicate + sodium fluoride dentifrice demonstrated intermediate performance, with no significant advantage over conventional NaF alone. Although SCP/NaF is recognized for managing dentin hypersensitivity, its protective effect on enamel under erosive–abrasive conditions remains inconsistent [10, 44, 47]. Factors such as short contact time, restricted ion release or limited surface precipitation may contribute to this variability [44, 47]. Previous investigations likewise reported modest efficacy of SCP/NaF in preventing microhardness loss and structural degradation [15, 16, 20]. More favorable outcomes have been described in protocols with prolonged exposure or increased ion availability, suggesting that the performance of this technology is highly dependent on experimental parameters and substrate characteristics [12, 35, 38]. While SCP‐based formulations remain clinically valuable for hypersensitivity management [44, 48], their effectiveness for erosion prevention appears limited under the present in vitro conditions and warrants confirmation in future in situ studies.

Surface roughness analysis showed no significant increases attributable to any of the tested dentifrices. Within the limits of the model, this suggests that none of the formulations produced additional topographic changes that could increase susceptibility to mechanical wear or facilitate plaque accumulation or extrinsic staining [6, 21]. The QLF findings reinforced this interpretation: BAS/NaF and SnF maintained fluorescence values closer to baseline [31], consistent with profilometric evidence of lower vertical enamel loss [12, 16, 19, 37]. Together, the optical and topographical outcomes indicate that these formulations provided a more effective barrier against erosive–abrasive degradation. This protection was reflected in smoother surfaces with fewer depressions and less profile distortion, supporting the interpretation that the protective effects of these dentifrices operate synergistically to preserve both surface integrity and subsurface mineral status [16, 33].

It is important to clarify that this is an in vitro study designed to simulate erosive–abrasive tooth wear rather than to reproduce the complex environment of the oral cavity. Although the present findings indicate the potential of bioactive ingredients to enhance enamel protection, the in vitro design limits direct clinical extrapolation. Variables such as brushing force, dietary patterns, salivary dynamics, and exposure frequency were necessarily standardized and may not fully reflect the variability encountered under clinical conditions. Additionally, nondestructive techniques capable of assessing the thickness of the subsurface softened enamel layer could further refine the interpretation of surface microhardness outcomes in future studies. Therefore, in situ and clinical studies are required to confirm whether these effects translate into clinically relevant and durable protection.

The use of deionized water for slurry preparation represents a deliberate methodological choice aimed at standardizing experimental conditions and isolating the intrinsic effects of the dentifrice formulations. Additionally, the pH of the toothpaste slurries was not independently measured; although slurry pH may influence dentifrice performance, the pH of diluted slurries does not fully represent the dynamic intraoral conditions during toothbrushing, which are modulated by salivary buffering, dilution, and clearance. Although fluoride efficacy may be enhanced in calcium‐rich environments, mineral‐containing slurries could introduce additional sources of calcium and phosphate, potentially confounding the interpretation of bioactive mechanisms. In the present protocol, mineral availability was ensured during the remineralization phases through the use of artificial saliva. Future studies employing mineralized slurries or in situ models may further elucidate the role of calcium–fluoride interactions under erosive–abrasive conditions.

## AUTHOR CONTRIBUTIONS

**Conceptualization**: Oliveira AFB, Sampaio FC. **Methodology**: Oliveira AFB, Sampaio FC, Cunha JL, Forte AG. **Investigation**: Cunha JL, Forte AG, Souza EBG, Paiva MA, Bacelar D. **Data curation**: Cunha JL, Forte AG, Souza EBG, Paiva MA, Bacelar D. **Formal analysis**: Oliveira AFB, Cunha JL, Forte AG, Souza EBG. **Project administration**: Oliveira AFB, Sampaio FC, **Supervision**: Oliveira AFB, Sampaio FC. **Validation**: Oliveira AFB, Sampaio FC. **Visualization**: Cunha JL, Forte AG, Souza EBG, Paiva MA, Bacelar D. **Writing—original draft**: Cunha JL, Forte AG. **Writing—review and editing**: Oliveira AFB.

## CONFLICT OF INTEREST STATEMENT

The authors declare no conflicts of interest.

## Data Availability

The datasets generated and analyzed during the current study are available from the corresponding author on reasonable request.
